# First-in-human infection imaging with ^89^Zr-labelled leukocytes and comparison of scan quality with [^99m^Tc]Tc-HMPAO-labelled leukocytes

**DOI:** 10.3389/fnume.2024.1426650

**Published:** 2024-07-22

**Authors:** Maryke Kahts, Beverley Summers, Akhona Nkokheli Ndlela, Aadil Gutta, Phumudzo Nemutaduni, Andrew More, Aman Parsoo, Thomas Ebenhan, Jan Rijn Zeevaart, Omer Aras, Mike Machaba Sathekge

**Affiliations:** ^1^ School of Pharmacy, Sefako Makgatho Health Sciences University, Ga-Rankuwa, South Africa; ^2^ Nuclear Medicine Department, Dr. George Mukhari Academic Hospital, Ga-Rankuwa, South Africa; ^3^ School of Medicine, Sefako Makgatho Health Sciences University, Ga-Rankuwa, South Africa; ^4^ Radiology Department, Dr. George Mukhari Academic Hospital, Ga-Rankuwa, South Africa; ^5^ Nuclear Medicine Research Infrastructure (NuMeRI) and Department of Nuclear Medicine, Steve Biko Academic Hospital, University of Pretoria, Pretoria, South Africa; ^6^ Radiochemistry, The South African Nuclear Energy Corporation, Pelindaba, South Africa; ^7^ DST/NWU, Preclinical Drug Development Platform, North-West University, Potchefstroom, South Africa; ^8^ Department of Radiology, Memorial Sloan Kettering Cancer Center, New York, NY, United States; ^9^ Department of Radiology, AMRIC Health, New York, NY, United States

**Keywords:** infection imaging, inflammation, [^99m^Tc]Tc-HMPAO, labelled leukocytes, WBC scan, zirconium-89, SPECT, PET/CT

## Abstract

**Introduction:**

Nuclear medicine infection imaging is routinely performed with the use of leukocytes radiolabelled with technetium-99m hexamethylpropyleneamine oxime ([^99m^Tc]Tc-HMPAO) and single-photon emission computed tomography (SPECT). Positron emission tomography (PET) is more sensitive than SPECT and results in higher-quality images. Zirconium-89 (^89^Zr) is a positron emitter with a half-life of 78.4 h, which translates to the biological half-life and slow biodistribution of intact cells and allows delayed PET imaging for more accurate biodistribution of the labelled leukocytes to infection foci. A first-in-human study with [^89^Zr]Zr-oxine-leukocytes in four healthy volunteers was reported in 2022. Our first-in-human study utilising the cell surface labelling approach aimed to image infection in patients with the use of ^89^Zr-labelled leukocytes, using *p*-isothiocyanatobenzyl-desferrioxamine B (Df-Bz-NCS) as a bifunctional chelating agent, and to compare the scan quality and biodistribution of [^89^Zr]Zr-Df-Bz-NCS-labelled leukocytes on PET images to SPECT images obtained with [^99m^Tc]Tc-HMPAO-labelled leukocytes.

**Methods:**

Leukocytes were isolated from whole-blood samples of eight patients with clinically and/or radiologically confirmed infection. Isolated leukocytes were labelled with [^99m^Tc]Tc-HMPAO according to standardised methods, and [^89^Zr]Zr-Df-Bz-NCS according to our previously published radiolabelling method. Whole-body SPECT imaging was performed 2 and 18 h post injection of [^99m^Tc]Tc-HMPAO-labelled leukocytes, and whole-body PET/CT was performed 3 and 24 h post injection of [^89^Zr]Zr-Df-Bz-NCS-labelled leukocytes in seven patients.

**Results:**

Successful [^89^Zr]Zr-Df-Bz-NCS-leukocyte labelling was achieved. High labelling efficiencies were obtained (81.7% ± 3.6%; *n* = 8). A mean high viability of [^89^Zr]Zr-Df-Bz-NCS-labelled leukocytes was observed (88.98% ± 12.51%). The [^89^Zr]Zr-Df-Bz-NCS-leukocyte labelling efficiency was not significantly affected by the white blood cell count of the patient. The performance of [^99m^Tc]Tc-HMPAO- and [^89^Zr]Zr-Df-Bz-NCS-labelled leukocytes, in terms of the ability to accurately detect infection, were similar in two out of seven patients, and [^99m^Tc]Tc-HMPAO-labelled leukocytes outperformed [^89^Zr]Zr-Df-Bz-NCS-labelled leukocytes in one patient with femoral osteomyelitis. However, in two cases of pulmonary pathology, [^89^Zr]Zr-Df-Bz-NCS-labelled leukocytes demonstrated improved pathological uptake. No skeletal activity was observed in any of the patients imaged with [^89^Zr]Zr-Df-Bz-NCS-labelled leukocytes, illustrating the *in vivo* stability of the radiolabel.

**Discussion:**

Although the [^89^Zr]Zr-Df-Bz-NCS-leukocyte labelling aspect of this study was noteworthy, infection imaging did not yield convincingly positive results due to the pulmonary trapping of intravenously administered [^89^Zr]Zr-Df-Bz-NCS-labelled leukocytes.

## Introduction

Nuclear medicine infection imaging is routinely performed with leukocytes radiolabelled with technetium-99m hexamethylpropyleneamine oxime ([^99m^Tc]Tc-HMPAO), indium-111 oxine ([^111^In]In-oxine), or indium-111 tropolone ([^111^In]In-tropolone) (1). Technetium-99m and indium-111 are both single-photon emitting radionuclides, allowing imaging via single-photon emission computed tomography (SPECT) (2–6). Radiolabelled leukocytes are sensitive for the detection of infection and inflammatory processes as they localise in infectious and inflammatory foci via the normal immune response, allowing imaging of these pathophysiological processes (7–9).

Compared to technetium-99m, which has a half-life of 6 h, indium-111 has a longer half-life of 67.2 h, allowing delayed imaging, which, in turn, allows more time for the radiolabelled leukocytes to distribute to infectious and inflammatory foci with resultant higher target-to-background ratios on imaging (4, 10, 11). However, technetium-99m possesses better physical characteristics (140 keV gamma emission and 6 h half-life), is more widely available at a lower cost, and results in a lower radiation burden to the patient, hence the more frequent use of [^99m^Tc]Tc-HMPAO-labelled leukocytes over [^111^In]oxine/tropolone-labelled leukocytes (3, 4).

In recent years, researchers have been investigating positron-emitting radionuclides to apply in positron emission tomography (PET) infection imaging, as PET is more sensitive than SPECT and results in higher-quality images (6, 12). Fluorine-18 fluorodeoxyglucose ([^18^F]FDG) is a PET radiopharmaceutical that is widely used in PET infection imaging, especially for fever of unknown origin, but also for a variety of other infectious diseases (13–15). The fact that [^18^F]FDG provides the opportunity for single-day imaging is noteworthy, as single-day imaging is far superior to any procedures that require two visits to the hospital (as is the case for radiolabelled leukocyte imaging). However, as radiolabelled leukocytes remain the gold standard for the imaging of various infections, [^18^F]FDG has been investigated as a radiolabel for leukocytes (16–19). A major disadvantage of [^18^F]FDG-labelled leukocytes is the short half-life of fluorine-18, only 109.7 min, which does not allow delayed imaging, that is often required for the accurate diagnosis of infection (3–5, 10, 20–21).

Zirconium-89 (^89^Zr) is a positron-emitting radionuclide with a half-life of 78.4 h, which translates to the biological half-life and slow biodistribution of intact cells and whole antibodies, and allows delayed PET imaging for up to 3 weeks after injection (4, 20, 22–27). Zirconium-89 decays via both positron emission (23%) and electron capture (77%), accompanied by a 909-keV gamma emission (22). Although fluorine-18 is a pure positron emitter and thus seems superior to zirconium-89, the difference in the energies of the 511 keV photons and the 909 keV gamma rays emitted during zirconium-89 decay prevents interference with the detection of the coincident photons resulting from the annihilation reaction in PET imaging (22, 28, 29). Free zirconium-89 is a bone-seeking agent, as it mimics iron and is chelated by hydroxyapatite on the bone surface (22).

Correspondingly, over the last decade, the labelling of leukocytes with zirconium-89 for PET imaging of infection has sparked some interest. Most recently, leukocyte labelling with [^89^Zr]Zr(oxinate)_4_ has been performed by Man et al. (20) and Massicano et al. (26). The [^89^Zr]Zr(oxinate)_4_ retention in leukocytes follows the same principle as [^111^In]In-oxine-labelled leukocytes, where a lipophilic complex is formed that can passively diffuse across the cell membrane and dissociates intracellularly for the radiolabel to become trapped within the cells. Labelling efficiencies achieved with this labelling approach ranged from approximately 20%–55% and high leukocyte viability after labelling was reported [67.05% ± 4.58% (26) and 99.4% ± 0.3% (20)]. A first-in-human study with [^89^Zr]Zr-oxine-labelled leukocytes in four healthy volunteers was undertaken by Lapi et al. (24), with promising results in terms of patient safety and biodistribution of [^89^Zr]Zr-oxine-labelled leukocytes.

To date, the majority of successful labelling attempts with zirconium-89 utilised conjugates of desferrioxamine B (DFO) as chelator for the [^89^Zr]Zr^4+^ metal ions (22, 28). Success was achieved especially with p-isothiocyanatobenzyl-desferrioxamine B (Df-Bz-NCS), which allows binding to primary amine groups of cell surface proteins (23, 30). Df-Bz-NCS has mainly been applied in the labelling of antibodies with zirconium-89 for immuno-PET studies (28). Most recently, Bansal et al. (23) labelled leukocytes with zirconium-89 via Df-Bz-NCS and reported labelling efficiencies of 22.77 ± 5.02% (*n* = 3), with high leukocyte viability of approximately 90%–95% after labelling. They further investigated the biodistribution of [^89^Zr]Zr-Df-Bz-NSC-labelled leukocytes in healthy athymic mice and found significantly high accumulation in the liver and spleen at 4 h, 2 days, 4 days, and 7 days post injection, with no skeletal uptake.

Our previous work investigated and optimised the cell surface labelling approach, using Df-Bz-NCS, to label human leukocytes with zirconium-89, and studied the biodistribution of [^89^Zr]Zr-Df-Bz-NCS-labelled leukocytes in a healthy mouse model (31). We achieved a mean labelling efficiency of 57% ± 12.46%, in the range of 46%–87%, over 10 labelling attempts. The normal biodistribution of the [^89^Zr]Zr-Df-Bz-NCS-labelled leukocytes was observed as high accumulation in the lungs and gradual uptake in the liver, with no accumulation in bones. Here, we report a first-in-human study aimed to image infection in patients with the use of ^89^Zr-labelled leukocytes, using Df-Bz-NCS as a bifunctional chelating agent, and to compare the scan quality and biodistribution of PET images obtained with [^89^Zr]Zr-Df-Bz-NCS-labelled leukocytes to SPECT images obtained with [^99m^Tc]Tc-HMPAO-labelled leukocytes.

## Materials and methods

### Study design

The study was prospective, comparative, and descriptive. The study was conducted at the Nuclear Medicine Department of a tertiary hospital in Gauteng, South Africa. Ethical approval was obtained from the institutional review board at the Sefako Makgatho Health Sciences University (Ref no. SMUREC/P/21/2017) and written informed consent was obtained from the study participants.

### Patient cohort

Eight patients were recruited for the study. All patients were receiving antibiotic treatment at the time of the study, which was commenced within 1–7 days before their recruitment for this study. The patient demographics are summarised in Table 1.

**Table 1:** Patient demographics (*n* = 8).

Patient No.	Biological sex	Age (years)	WBC count on first day of enrolment (High/normal/low)	Diagnosis
1	Male	63	Normal	Pneumonia
2	Male	32	High	Pneumonia
3	Male	60	High	Pneumonia
4	Male	75	Low	Abscess in the left hand
5	Female	27	Normal	Pneumonia
6	Male	44	High	Pneumonia
7	Male	38	Normal	Chronic osteomyelitis in the right distal femur
8	Male	30	High	Suspected infective endocarditis

Unfortunately, one patient who was diagnosed with severe pneumonia and on permanent supplemental oxygen support died during the course of the study. Imaging could not be completed for this patient.

### Preparation of the work area

All radiopharmaceutical labelling and preparation procedures were performed in a shielded Class II Biohazard Safety Cabinet, with GMP grade A vertical laminar flow over the complete working area (SAFEFLOW L30; Tema Sinergie, Faenza, Ravenna, Italy). The cabinet was switched on at least 15 min before commencing the work in the cabinet and disinfected with 70% isopropyl alcohol before and after each labelling and radiopharmaceutical preparation procedure. All required items were disinfected with 70% isopropyl alcohol before transfer into the laminar airflow cabinet. All labelling and radiopharmaceutical preparation procedures were performed by a qualified radiopharmacist with experience in the leukocyte labelling methods utilised.

### Leukocyte separation from whole blood

Peripheral whole-blood samples (45 ml) were drawn from eight patients with confirmed infection, into 10 BD Vacutainer® citrate tubes (#: 369714; Becton, Dickinson and Company, Franklin Lakes, NJ, USA) for each patient. Each sample was divided into two 50-ml Falcon tubes with frits (Greiner Bio-One, Kremsmünster, Austria), with each tube containing 15 ml of sterile-filtered Lymphocytes Separation Media, Density 1.077 g/ml (Capricorn Scientific GmbH, Ebsdorfergrund, Germany), and the sample was centrifuged at 800 g for 15 min to isolate the mononuclear layers (Heraeus™ Megafuge™ 8 centrifuge; Thermo Fisher Scientific, Waltham, MA, USA). The isolated mononuclear layers were removed with a plastic Pasteur pipette, transferred to a clean 50-ml Falcon tube, and washed twice with 10 ml of phosphate-buffered saline (PBS, pH 7.4; Gibco, Billings, MT, USA) (centrifuged at 250 g for 10 min each time). The washed cells were resuspended in 500 µl PBS for labelling.

### Determination of cell viability and count before and after labelling

Before labelling commenced, a cell viability test and cell count were performed to determine whether the isolated leukocytes were still functional. In performing these tests, a 20-µl sample of the isolated leukocytes was diluted with 20 µl of VS™ Cellometer AOPI Staining Solution-S (Nexcelom Bioscience LLC, Lawrence, MA, USA). After dilution, 20 μl of the sample was pipetted into the SD100 Counting Chamber (Nexcelom Bioscience LLC, Lawrence, MA, USA), the counting chamber was inserted into the slot on the cytometer and cell viability testing and cell counting commenced using the Cellometer® K2 Image Cytometer (Nexcelom Bioscience LLC, Lawrence, MA, USA). The cell viability test was repeated after leukocyte labelling for each labelling attempt to assess whether the labelled leukocytes were still functional before administration to the patients.

### [^99m^Tc]Tc-HMPAO-leukocyte labelling

To compare the utility of [^89^Zr]Zr-Df-Bz-NCS-labelled leukocytes with [^99m^Tc]Tc-HMPAO-labelled leukocytes for infection imaging, the labelling of leukocytes with [^99m^Tc]Tc-HMPAO was performed according to the European Association of Nuclear Medicine (EANM) guidelines authored by De Vries et al. (32), and is described in detail below. [^99m^Tc]Tc-HMPAO-leukocyte labelling was performed for seven patients in this study. The remaining patient was scheduled for a routine [^99m^Tc]Tc-HMPAO-leukocyte scan at the Nuclear Medicine Department of the primary research site, and [^99m^Tc]Tc-HMPAO-leukocyte labelling was performed by a centralised radiopharmacy. This patient was recruited for the study due to his diagnosis (osteomyelitis in the femur).

#### Technetium-99m elution

Na[^99m^Tc]TcO_4_ elution was performed according to the EANM guidelines stating that a fresh eluate from a generator that was eluted within the previous 24 h is required for [^99m^Tc]Tc-HMPAO labelling (32). A molybdenum-99/technetium-99m generator [NovaTec-P Tc-99m Generator, Nuclear Technology Products (NTP) Radioisotopes SOC Ltd., Pelindaba, South Africa] located in the Nuclear Medicine Department of the Dr George Mukhari Academic Hospital was used for Na[^99m^Tc]TcO_4_ elution between 11:20 a.m. and 12:04 p.m. every day that [^99m^Tc]Tc-HMPAO-leukocyte labelling was performed for our study. The generator was routinely eluted for scheduled SPECT/CT procedures between 7:30 and 8:00 every morning, i.e., ∼4 h before elution for [^99m^Tc]Tc-HMPAO-leukocyte labelling. For each elution, a saline vial (sodium chloride 0.9% solution, 20 ml) and elution (vacuum) vial (Eluvial, 30 ml) (both NTP Radioisotopes SOC Ltd., Pelindaba, South Africa) were used. After the metal safety discs were removed from the vial caps, the vial stoppers were sanitised with an isopropyl alcohol swab and allowed to dry. The inlet spike of the generator was then uncovered, and the saline vial was attached by piercing the generator inlet spike into the vial. The elution vial was then inserted into a lead shield and attached to the outlet spike of the generator. Once the bubbling observed in the elution vial had subsided, the elution vial was removed from the generator and the outlet spike was capped with a sterile vacuum vial. After elution, the technetium-99m activity eluted was measured with a dose calibrator (Atomlab™ 500, Biodex Medical Systems, Shirley, NY, USA) and recorded.

#### [^99m^Tc]Tc-HMPAO preparation

To prepare [^99m^Tc]Tc-HMPAO, an HMPAO vial (Exametazime 500 μg, Medi-Exametazim #ME-211204; Medi-Radiopharma Ltd., Hungary) was placed in a lead shield and the rubber septum was sanitised with an isopropyl alcohol swab and allowed to dry. Then, with the use of a 10-ml syringe, 5 ml of sterile, freshly eluted Na[^99m^Tc]TcO_4_ was injected into the HMPAO vial, and 5 ml of gas was removed from the space above the solution before withdrawing the syringe from the vial to normalise the pressure in the vial. The Na[^99m^Tc]TcO_4_ eluate was added to the HMPAO kit vial within 10 min of elution, in keeping with the EANM guidelines stating that a fresh eluate (preferably within 30 min of elution) is required for [^99m^Tc]Tc-HMPAO labelling (32). The shielded vial was gently inverted for 10 s to ensure complete dissolution of the powder. The solution was then incubated for 5 min at bench top temperature to allow the formation of the [^99m^Tc]Tc-HMPAO complex.

#### Quality control of the [^99m^Tc]Tc-HMPAO complex

Before leukocyte radiolabelling commenced, the radiochemical purity of the prepared [^99m^Tc]Tc-HMPAO complex was determined via the miniaturised chromatography method as recommended by the European Pharmacopoeia (33). Glass microfiber chromatography paper impregnated with silica gel (ITLC-SG) (Agilent Technologies, Santa Clara, CA, USA) was used as the stationary phase and two development chambers were prepared – the first with methyl ethyl ketone (MEK; Butanone, Chemically Pure, Rochelle Chemicals, Johannesburg, South Africa) as mobile phase and the second with normal saline [0.9% w/v sodium chloride; B. Braun (Pty) Ltd., Johannesburg, South Africa] as mobile phase. A 5-µl sample of the prepared [^99m^Tc]Tc-HMPAO complex was spotted on the origin (R_f_ = 0) of two ITCL-SG strips, the strips were placed in the development chambers and the development chambers were closed. After development, the strips were dried and the separation measured with a radiochromatographic scanner (Scan-RAM with Laura software, LabLogic Systems Limited, Sheffield, UK). With the use of MEK as the mobile phase, any secondary [^99m^Tc]Tc-HMPAO species and hydrolysed reduced technetium (HRTc) remained at the origin (R_f_ = 0), while the desired lipophilic primary [^99m^Tc]Tc-HMPAO species and free pertechnetate ([^99m^Tc]TcO_4_^−^) moved towards the solvent front (R_f_ = 0.8–1.0). The use of normal saline as mobile phase, resulted in the movement of [^99m^Tc]TcO_4_^−^ to the solvent front (R_f_ = 0.8–1.0), while all primary and secondary [^99m^Tc]Tc-HMPAO species, as well as HRTc remained at the origin (R_f_ = 0). The radiochemical purity of [^99m^Tc]Tc-HMPAO was then calculated by subtracting the peak formed at the solvent front of the strip developed in saline (i.e., the [^99m^Tc]TcO_4_^−^ peak) from the peak formed towards the solvent front of the strip developed in MEK (i.e., the desired primary [^99m^Tc]Tc-HMPAO species and [^99m^Tc]TcO_4_^−^ peak) to ultimately obtain the percentage of the desired primary [^99m^Tc]Tc-HMPAO complex present in the sample. A radiochemical purity of ≥80% is required by the European Pharmacopoeia (33).

#### Cell labelling

After determining the radiochemical purity of the prepared [^99m^Tc]Tc-HMPAO complex, to label leukocytes with [^99m^Tc]Tc-HMPAO, 1 ml of the prepared [^99m^Tc]Tc-HMPAO complex was added to the mixed leukocyte suspension (500 µl) and the mixture was incubated for 15–20 min at room temperature, with periodic, gentle swirling of the cell suspension to prevent sedimentation of the cells. After incubation, 10 ml of PBS (pH 7.4) was added and the suspension was centrifuged at 150 g for 5 min. The supernatant was removed with a plastic Pasteur pipette and transferred to a clean 15-ml Falcon tube. The cell pellet was gently resuspended in 5 ml of PBS and centrifuged again at 150 g for 5 min. The supernatant was added to the previous supernatant in the 15-ml Falcon tube and the activity of the combined supernatants was measured and recorded. The activity of the labelled cells was also measured and recorded, and the labelling efficiency was calculated with the following formula (4):

$$\mathrm{Labelling}\;\mathrm{effiency}\;(\%)=\frac{\mathrm{Activity}\;\mathrm{in}\;\mathrm{the}\;\mathrm{cells}\;(MB_{q})}{\mathrm{Activity}\;\mathrm{in}\;\mathrm{the}\;\mathrm{combined}\;\mathrm{supernatants}\;(MB_{q})}+\mathrm{Activity}\;\mathrm{in}\;\mathrm{the}\;\mathrm{cells}\;(MB_{q})\times 100$$

The labelled cells were gently resuspended in 3 ml PBS and the patient dose was dispensed.

### [^89^Zr]Zr-Df-Bz-NCS-leukocyte labelling

To prepare [^89^Zr]Zr-Df-Bz-NCS-labelled leukocytes, [^89^Zr]Zr-oxalate in oxalic acid imported by Biocom Africa (Pty) Ltd. (Centurion, South Africa) from the Netherlands (BV Cyclotron VU, Amsterdam, The Netherlands) was utilised.

#### Zirconium-89 neutralisation

Of the imported [^89^Zr]Zr-oxalate, various volumes (110–246 µl) and activities (82.880–150.590 MBq) were neutralised as follows: the [^89^Zr]Zr-oxalate solution to be used for labelling was added to a 15-ml Falcon tube (Greiner Bio-One, Kremsmünster, Austria) and the activity was measured in a dose calibrator (Atomlab™ 500, Biodex Medical Systems, Shirley, NY, USA) and recorded. An equal volume of sodium bicarbonate solution (7.5%, sterile-filtered, BioReagent, suitable for cell culture, Sigma-Aldrich, St Louis, MO, USA) was added to the [^89^Zr]Zr-oxalate solution and gently mixed until the bubbling subsided.

#### [^89^Zr]Zr-Df-Bz-NCS chelation

To prepare [^89^Zr]Zr-Df-Bz-NCS, a sufficient volume of dimethyl sulfoxide (DMSO) (Life Technologies Corporation, Carlsbad, CA, USA) was gently mixed with a specific amount of Df-Bz-NCS (Cas# 1222468-90-7; Macrocyclics, Inc., Plano, TX, USA) to yield an oversaturated Df-Bz-NCS in DMSO solution with a concentration of 0.075 mg/µl and the resulting mixture was centrifuged at 1,000 rpm for 1 min to form a pellet. The pellet consisted of the excess Df-Bz-NCS not dissolved in DMSO. The supernatant of the DMSO-Df-Bz-NCS mixture, containing the dissolved Df-Bz-NCS, was removed without disturbing the pellet and added to the 15-ml Falcon tube containing neutralised [^89^Zr]Zr^4+^, followed by the addition of PBS. The pH of the solution was adjusted with sodium bicarbonate solution (7.5%, Sigma-Aldrich, St Louis, MO, USA) to obtain a final pH of 7–8.

#### Cell labelling

To label leukocytes with [^89^Zr]Zr-Df-Bz-NCS, washed leukocytes were added to the [^89^Zr]Zr-Df-Bz-NCS mixture and the activity was measured and recorded, followed by incubation of the mixture for 45 min at 37°C. After incubation, the [^89^Zr]Zr-Df-Bz-NCS-labelled cells were washed twice with 5 ml of PBS, where the supernatants were combined in a clean 15-ml Falcon tube. The activity of the combined supernatants and the [^89^Zr]Zr-Df-Bz-NCS-labelled cells were measured and recorded to determine the labelling efficiency (with the same labelling efficiency formula as used for [^99m^Tc]Tc-HMPAO-labelled cells). The [^89^Zr]Zr-Df-Bz-NCS-labelled cells were gently resuspended in 3 ml PBS for administration and the activity of the patient dose was measured and recorded.

### Imaging protocols

#### Radiopharmaceutical administration to human patients

Patients were administered [^99m^Tc]Tc-HMPAO-labelled leukocytes and [^89^Zr]Zr-Df-Bz-NCS-labelled leukocytes (on different days) via a 20- or 21-gauge needle and the activity left in the syringe after administration was measured and recorded to calculate the exact patient dose. The recommended dose of [^99m^Tc]Tc-HMPAO-labelled leukocytes to be administered to patients is 370–740 MBq, according to the EANM guidelines for the labelling of leukocytes with [^99m^Tc]Tc-HMPAO (32). Regarding the dose of [^89^Zr]Zr-Df-Bz-NCS-labelled leukocytes, the 909 keV gamma emitted by zirconium-89 limits the dose that can be administered to patients and must be taken into consideration to minimise the radiation exposure to the patient, thus the dose of [^89^Zr]Zr-Df-Bz-NCS-labelled leukocytes to be administered to the patients was minimised to 37–74 MBq and was dependant on the activity of zirconium-89 available for radiolabelling, as well as the radiolabelling efficiency.

After PET/CT imaging of the first three patients, cell clumping was identified as a factor affecting the distribution of the administered [^89^Zr]Zr-Df-Bz-NCS-labelled leukocytes. This was addressed with the addition of 6 µl (10 units/ml of patient dose) of heparin solution [Heparin Sodium Fresenius 5,000 IU/ml, 5 ml vial; Fresenius Kabi Manufacturing SA (Pty) Ltd., Midrand, South Africa] to the dose before it was administered to the patient. This cell clumping phenomenon did not occur with [^99m^Tc]Tc-HMPAO-labelled leukocytes.

#### Image acquisition

Imaging was performed using whole-body SPECT/CT after the administration of [^99m^Tc]Tc-HMPAO-labelled leukocytes and whole-body PET/CT after the administration of [^89^Zr]Zr-Df-Bz-NCS-labelled leukocytes.

Whole-body SPECT/CT (NM/CT 860 dual-head SPECT/CT system, GE HealthCare, Chicago, IL, USA) was performed 2 h after administration of the [^99m^Tc]Tc-HMPAO-labelled leukocytes and repeated 18 h after radiopharmaceutical administration. For both sets of images, patients were positioned supine with arms above the head and the mid-thighs to the base of the skull considered the area of interest. However, in patients with an area of interest outside the field of view, the area was imaged separately after the completion of whole-body SPECT/CT. Patients were positioned feet first and the scan direction was caudocranial to minimise counts from a full bladder during the scan via early imaging of the pelvic area.

A pre-set multiple-bed SPECT imaging protocol was used. The bed position was set to a height of 90.0 cm and a range of 111 cm with an overlap of 4.42 cm. The detectors were set to H mode, with a starting angle of 0°, and the camera rotation was set to a total of 360° angular range with a view angle of 6° and a total number of 60 views. The scan was set to 16 s step-and-shoot, with acquisition during motion between steps. The parameters were a 128 × 128 matrix size and a zoom of 1.0. SPECT was acquired first, followed by CT. The CT annotation was set to full scan with a display field of view of 50.0 cm and a matrix size of 512 × 512. The CT parameters were set to 12 kVp and 55 mAs.

Whole-body (base of skull to mid-thighs) PET/CT (Ingenuity TF PET/CT system; Philips Healthcare, Amsterdam, The Netherlands) was performed approximately 2 h after administration of the [^89^Zr]Zr-Df-Bz-NCS-labelled leukocytes and repeated approximately 24 h after radiopharmaceutical administration. In some cases, further delayed PET/CT was carried out, dependent on the availability and consent of the patient. Due to equipment malfunction at the primary research site, a Siemens Biograph 450 PET/CT system (Siemens Healthineers, Erlangen, Germany) located at the South African Nuclear Medicine Research Infrastructure/Steve Biko Academic Hospital, was also utilised to scan one participant.

Patients were positioned with arms above the head on the PET/CT camera bed and the glabellomeatal line was used for standardisation. The direction of the scan was caudocranial. We utilised the radionuclide window for iodine-124 (^124^I), as the PET/CT camera at the primary research site did not have the settings for zirconium-89 and iodine-124 shares a similar half-life. The scan speed for the initial PET/CT scan was set to 2 min per bed position and 3 min per bed position for the delayed imaging, with the number of beds varying dependent on the height of the patient. The CT component was a low-dose 5 × 5, with 100 kVp: 95 mAs, with a dose length product (DLP) of 345.8 and scan time of 13.9 s. The low-dose CT was used for attenuation correction and anatomical localisation. The standard whole-body PET parameters were used, i.e., 128 × 128 matrix, slice thickness of 2.00 mm, zoom of 1.00, and pixel size of 2–4 mm. An appropriate scatter correction was applied for 3D mode imaging. All quality control parameters were checked and corrected before interpretation.

#### Image interpretation

Two nuclear medicine physicians assessed SPECT and PET images independently, and one radiologist assessed CT images. Abnormal and increased radiopharmaceutical uptake on the white blood cell (WBC) scan, with persisting or increasing intensity with time, was considered to indicate a positive study for infection (3).

## Results

### [^99m^Tc]Tc-HMPAO-labelled leukocytes

#### Radiochemical purity of the [^99m^Tc]Tc-HMPAO complex

In general, high radiochemical purity of the [^99m^Tc]Tc-HMPAO complex before its utilisation for leukocyte labelling was achieved, with a mean of 91.70 ± 7.54% (*n* = 7). In one case. the radiochemical purity was 73.46%, i.e., less than 80%, as recommended by the European Pharmacopoeia (33). There was only a low degree of correlation observed between the activity of technetium-99m added to the HMPAO vial and the radiochemical purity of the [^99m^Tc]Tc-HMPAO complex (*r* = 0.291).

#### [^99m^Tc]Tc-HMPAO-leukocyte labelling efficiency

**Figure 1:**
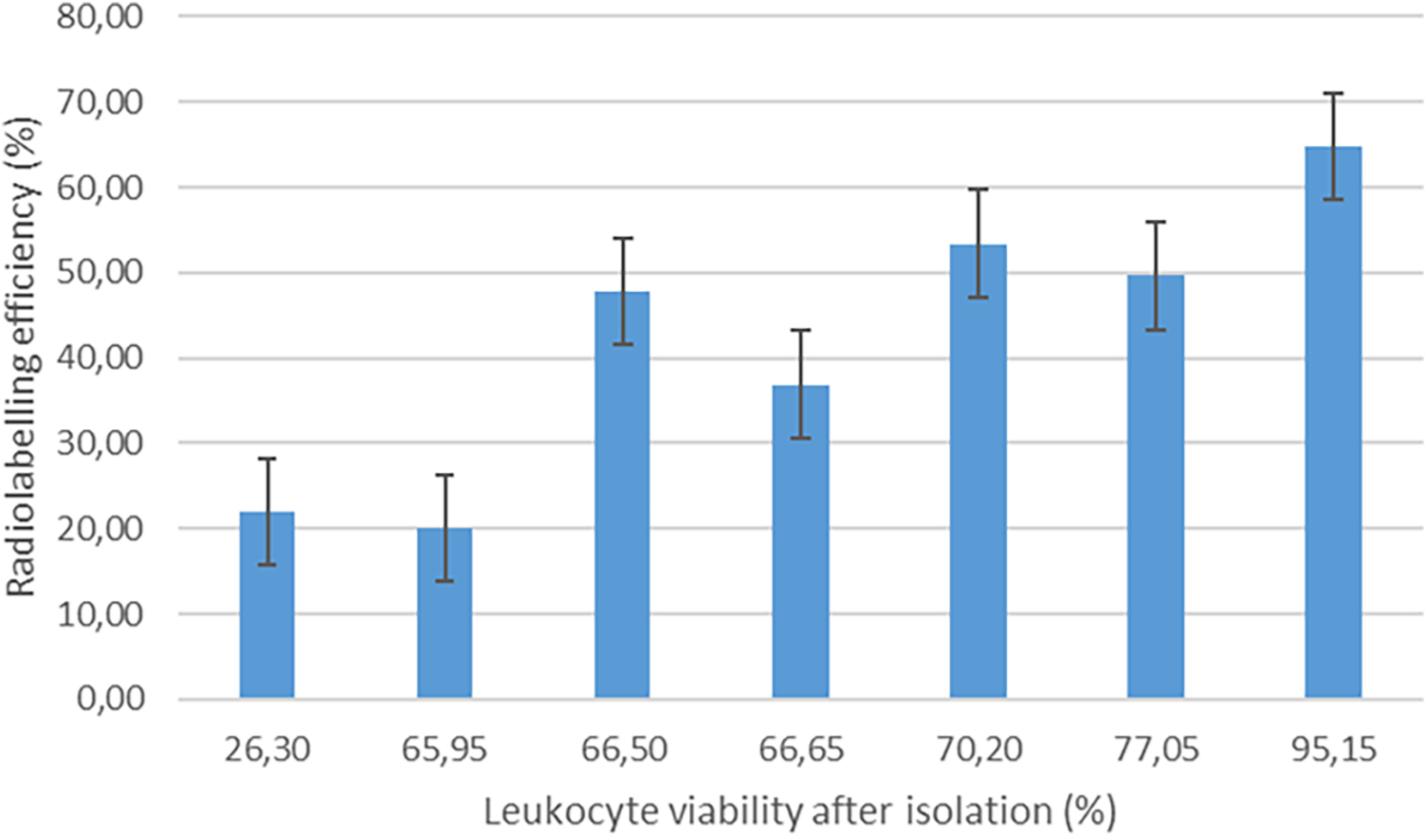
The effect of leukocyte viability after isolation on the labelling efficiency of [^99m^Tc]Tc-HMPAO-labelled leukocytes (*n* = 7).

The EANM guidelines for the labelling of leukocytes with [^99m^Tc]Tc-HMPAO state that [^99m^Tc]Tc-HMPAO mainly labels granulocytes (70%–80%) in a mixed leukocyte population (32). As mononuclear cells (i.e., lymphocytes and monocytes) were isolated for radiolabelling in this study, we expected a relatively low leukocyte labelling efficiency in comparison with the expected range of 40%–80% stated in the EANM guidelines (32). The mean [^99m^Tc]Tc-HMPAO-leukocyte labelling efficiency in this study was 42.06% ± 15.35%, with a range of 20%–64.74%. Interestingly, no significant correlation was observed between the radiochemical purity of [^99m^Tc]Tc-HMPAO and [^99m^Tc]Tc-HMPAO-leukocyte labelling efficiency (*r* = 0.102). There was only a moderate degree of correlation between white blood cell count and [^99m^Tc]Tc-HMPAO-leukocyte labelling efficiency (*r *= 0.370). The only factor that had a direct effect on the [^99m^Tc]Tc-HMPAO-leukocyte labelling efficiency was the viability of the isolated leukocytes before labelling (*r* = 0.773), as presented in Figure 1.

#### Leukocyte viability before and after [^99m^Tc]Tc-HMPAO labelling

**Figure 2:**
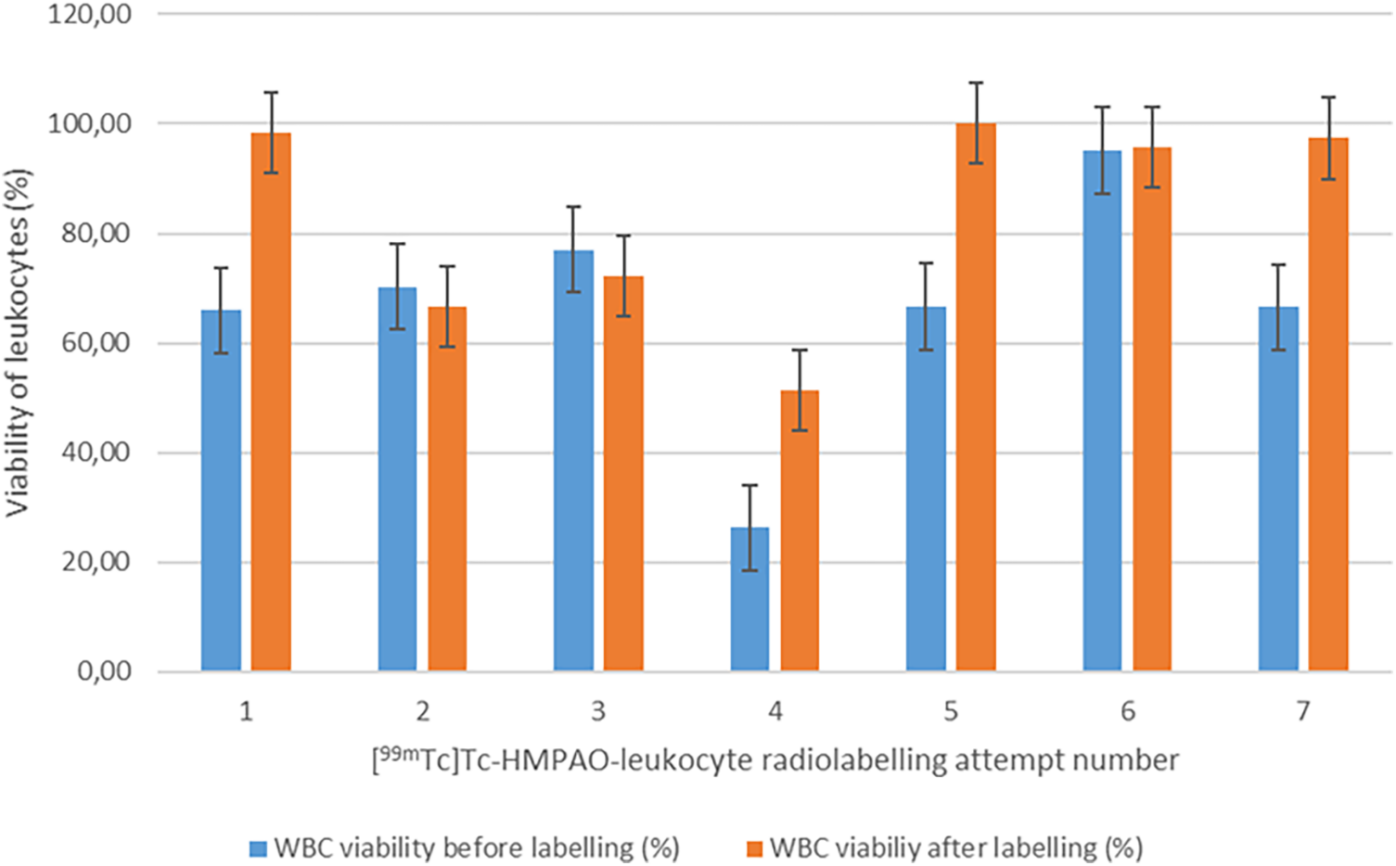
Viability of leukocytes before and after labelling with [^99m^Tc]Tc-HMPAO (*n* = 7).

To determine whether [^99m^Tc]Tc-HMPAO labelling affected the viability and resultant functionality of the leukocytes, the mean viability of the leukocytes directly after isolation from whole blood (66.83% ± 19.13%) was compared with the mean viability of leukocytes directly after labelling (83.12% ± 18.02%). Surprisingly, there was a significant increase in the viability of leukocytes after labelling (*p* = 0.03). We speculate that the increase in leukocyte viability after labelling may be due to the removal of dead leukocytes in the washing steps after labelling, which could result in a larger ratio of viable cells in the sample. The viability results before and after labelling are summarised in Figure 2.

### [^89^Zr]Zr-Df-Bz-NCS-labelled leukocytes

#### [^89^Zr]Zr-Df-Bz-NCS-leukocyte labelling efficiency and yield

Table 2 summarises the labelling results (labelling efficiency and yield) for all patients (*n* = 8), alongside the labelling parameters that could have affected the labelling efficiency and yield, i.e., the molar activity of [^89^Zr]Zr-Df-Bz-NCS, total reaction volume, and the leukocyte count and viability before labelling.

**Table 2:** Apparent molar activity of the [^89^Zr]Zr-Df-Bz-NCS complex (MBq/µmol), total reaction volume (ml), leukocyte count (cells/ml), and viability (%) before labelling, as well as the ^89^Zr-leukocyte radiolabelling efficiency (%) and yield (%) achieved for ^89^Zr labelling of leukocytes from eight patients with infection.

Patient No.	Molar activity (MBq/µmol)	Reaction volume (ml)	Leukocyte count (cells/ml)	Leukocyte viability (%)	Radiolabelling efficiency (%)	Radiolabelling yield (%)
1	31.22	2.94	1,560,000	61.00	87.4	72.92
2	36.71	2.57	7,055,000	79.35	84.0	73.48
3	32.50	3.12	356,000	84.70	79.0	60.63
4	51.68	2.74	42,000	66.70	78.2	70.35
5	43.19	2.86	6,700	50.00	82.5	76.77
6	45.36	3.41	1,475,000	95.95	75.6	68.06
7	43.84	2.30	764,300	95.65	82.4	71.61
8	48.44	2.51	3,820,000	80.15	84.2	71.25
Mean	41.62 ± 6.93	2.80 ± 0.3	1,884 875	76.69 ± 15.30	81.7 ± 3.6	70.63 ± 4.46

**Figure 3:**
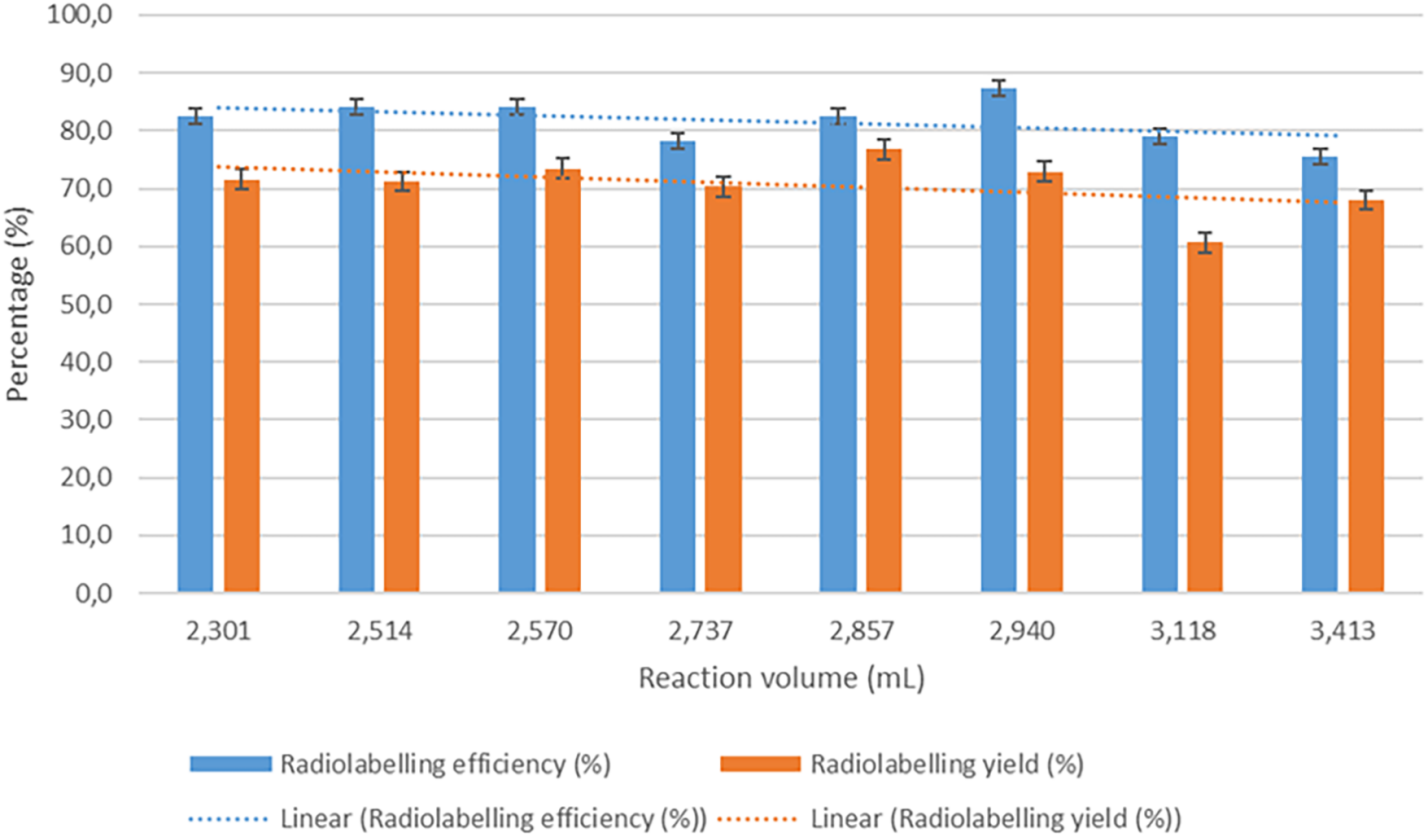
The effect of reaction volume (ml) on the labelling efficiency (%) and yield (%) of [^89^Zr]Zr-Df-Bz-NCS-labelled leukocytes (*n* = 8).

High and consistent [^89^Zr]Zr-Df-Bz-NCS-leukocyte labelling efficiencies were achieved, with a mean of 81.7% ± 3.6% and a range of 75.6%–87.4% (*n* = 8). [^89^Zr]Zr-Df-Bz-NCS labelling of leukocytes was performed with zirconium-89 activities in the range of 82.880–150.590 MBq (mean = 115.671 ± 21.177 MBq) and a mean [^89^Zr]Zr-Df-Bz-NCS apparent molar activity of 41.615 ± 6.933 MBq/µmol. There was a moderate correlation between the number of leukocytes and the [^89^Zr]Zr-Df-Bz-NCS-leukocyte labelling efficiency (*r* = 0.396), but no significant effect of the leukocyte viability on the radiolabelling efficiency was observed. There was a relatively negative effect of larger reaction volumes on the [^89^Zr]Zr-Df-Bz-NCS-leukocyte labelling efficiency (*r *= −0.539) and yield (*r* = −0.454), as illustrated in Figure 3.

#### Leukocyte viability before and after [^89^Zr]Zr-Df-Bz-NCS labelling

**Figure 4:**
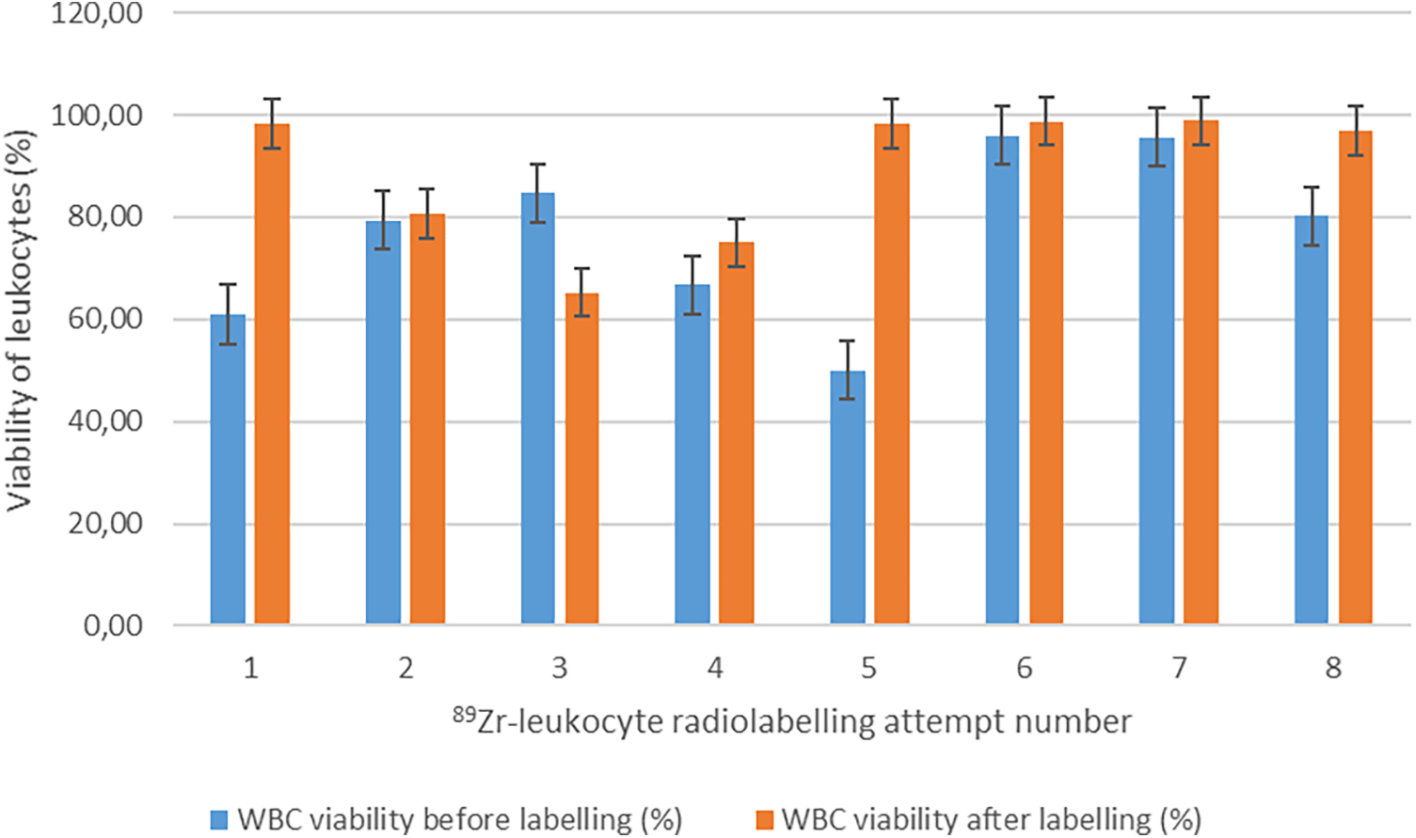
Viability of leukocytes before and after radiolabelling with [^89^Zr]Zr-Df-Bz-NCS (*n* = 8).

To determine whether [^89^Zr]Zr-Df-Bz-NCS labelling affected the viability and resultant functionality of the leukocytes, the mean viability of the leukocytes directly after isolation from whole blood (76.69% ± 15.30%) was compared with the mean viability of the leukocytes directly after labelling (88.98% ± 12.51%). As with [^99m^Tc]Tc-HMPAO labelling, a slight increase in the mean viability of leukocytes after ^89^Zr labelling was observed (*p* = 0.075), possibly due to the removal of dead leukocytes after labelling was completed. Another explanation for the slightly increased viability of leukocytes after radiolabelling may be the 45-min incubation period for radiolabelling to occur, which may have provided the time and conditions required for the leukocytes to recover from the isolation procedure. The viability results before and after ^89^Zr labelling are summarised in Figure 4.

### Radiation burden to the patient

A summary of the estimated whole-body effective doses received by the patients recruited for this study is provided in Table 3. The estimated whole-body effective dose from [^99m^Tc]Tc-HMPAO- and [^89^Zr]Zr-Df-Bz-NCS-labelled leukocytes were calculated from mean doses reported by De Vries et al. (32) and Yoon et al. (28), respectively.

**Table 3:** Estimated whole-body effective doses received by patients to whom [^99m^Tc]Tc-HMPAO-labelled leukocytes and [^89^Zr]Zr-Df-Bz-NCS-labelled leukocytes were administered for SPECT and PET/CT infection imaging, respectively (*n* = 8).

Patient No.	[^99m^Tc]Tc-HMPAO-labelled leukocytes		[^89^Zr]Zr-Df-Bz-NCS-labelled leukocytes		Total calculated whole-body effective dose (mSv)
	Administered dose (MBq)	Estimated whole-body effective dose (mSv)	Administered dose (MBq)	Estimated whole-body effective dose (mSv)	
1	231.620	2.548	58.460	31.568	34.116
2	386.650	4.253	29.785	16.084	20.337
3	531.690	5.849	39.035	21.079	26.927
4	323.010	3.553	61.420	33.167	36.720
5	429.940	4.729	65.490	35.365	40.094
6	593.850	6.532	75.961	41.019	47.551
7	270.100	2.971	43.068	23.257	26.228
8	523.550	5.759	48.544	26.214	31.973
Mean	411.301 ± 123.182	4.524 ± 1.355	52.720 ± 14.299	28.469 ± 7.722	32.993 ± 8.072

### Non-pathological radiopharmaceutical accumulation

*SPECT*: Early reconstructed images of all patients revealed low-grade diffuse uptake in the lung fields bilaterally, likely due to the margination of radiolabelled leukocytes. Accumulation of activity in the urinary bladder, as well as faint uptake in the kidneys in some cases, was observed.

**Figure 5:**
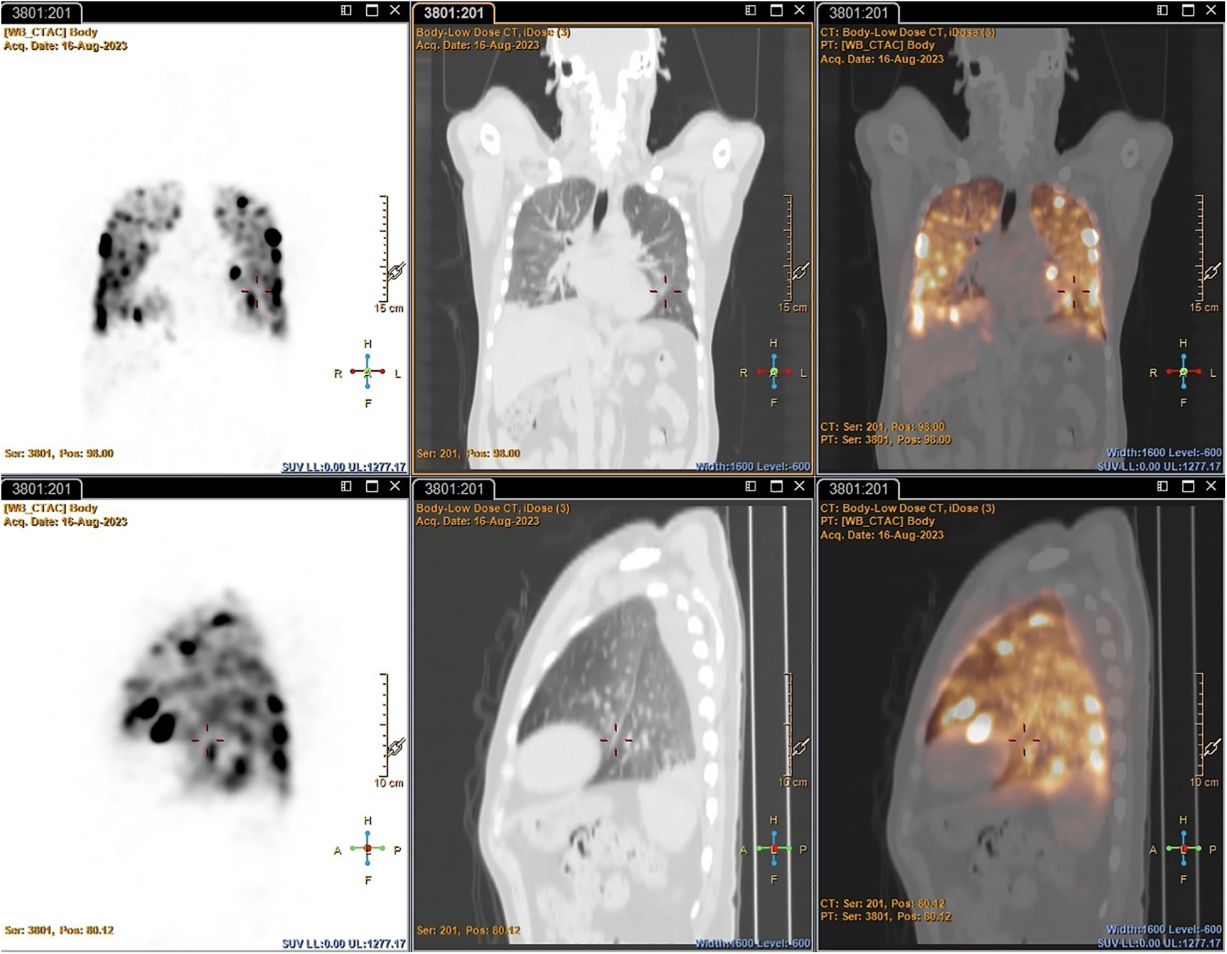
PET/CT images illustrating non-pathological focal pulmonary accumulation of ^89^Zr-labelled leukocytes in a patient with pneumonia (Patient 1).

*PET*: Both early and delayed images of all patients revealed either multiple foci of varying size and intensity in both lung fields (before the introduction of heparin to the dose) or as diffuse uptake in both lung fields (after the introduction of heparin to the dose), likely due to the accumulation of [^89^Zr]Zr-Df-Bz-NCS-labelled leukocytes that did not necessarily correspond with changes in the lung fields observed on the CT component. The foci of increased uptake were likely artefactual due to cell clumping/aggregation, rather than pathological. See Figure 5 for an example of non-pathological pulmonary focal accumulation of ^89^Zr-labelled leukocytes. Low-grade activity was noted in the genitourinary tract of all patients on the PET images.

### Nuclear medicine infection imaging reports

A summary of the findings of and correlation between the SPECT images obtained with [^99m^Tc]Tc-HMPAO-labelled leukocytes and the PET images obtained with ^89^Zr-labelled leukocytes for seven patients with infection is presented in Table 4.

**Table 4:** A summary of the findings of and correlation between SPECT images obtained with [^99m^Tc]Tc-HMPAO-labelled leukocytes and PET images obtained with ^89^Zr-labelled leukocytes for seven patients with infection.

Patient No.	Diagnosis	Findings on CT	Evidence of pathology on early SPECT? (Yes/No)	Evidence of pathology on delayed SPECT? (Yes/No)	Evidence of pathology on early PET? (Yes/No)	Evidence of pathology on delayed PET? (Yes/No)	Correlation between SPECT and PET
1	Pneumonia	Pulmonary oedema, cardiomegaly, possible congestive cardiac failure, possible superimposed lung infection	Yes	Not reportable	Yes	Yes	Increased uptake in parenchymal changes in the lung bases bilaterally on both SPECT and PET
2	Pneumonia	Right segmental consolidation with features of acute pulmonary infection	Yes (mild uptake)	No	Yes	Yes	Improved pathological uptake on PET compared to SPECT
3	Pneumonia	The distribution of the opacities noted in both lung fields are suggestive of bacterial pneumonia	No	Yes	Yes	Yes	Similar uptake in the right lung base which corresponded to pneumonic changes on the CT component of the study. Pathological uptake observed in the left lung with PET (low diagnostic confidence), but not with SPECT
4	Abscess in the left hand	Fluid collection in the dorsum of the left hand, with associated soft tissue swelling and fat stranding	Yes	Yes	Yes	Yes	Similar uptake in the left distal forearm//left hand
5	Pneumonia	Right bronchopneumonia—possibly bacterial infection or reactivated tuberculosis	No	No	No	No	No significant uptake in the parenchymal changes evident on CT
6	Chronic osteomyelitis in the right femur	Imaging features are suggestive of chronic right femoral osteomyelitis with involucrum formation	Yes	Yes	No	No	Positive SPECT study for chronic infection in the right distal femur, but non-diagnostic on PET
7	Suspected infective endocarditis and lung pathology	Cavitating pneumonia or tuberculosis—possibly septic pulmonary emboli	No	No	No	No	No evidence of radiolabelled leukocyte-avid endocarditis. No significant uptake in the CT evident cavitating infective process in the lungs

**Figure 6:**
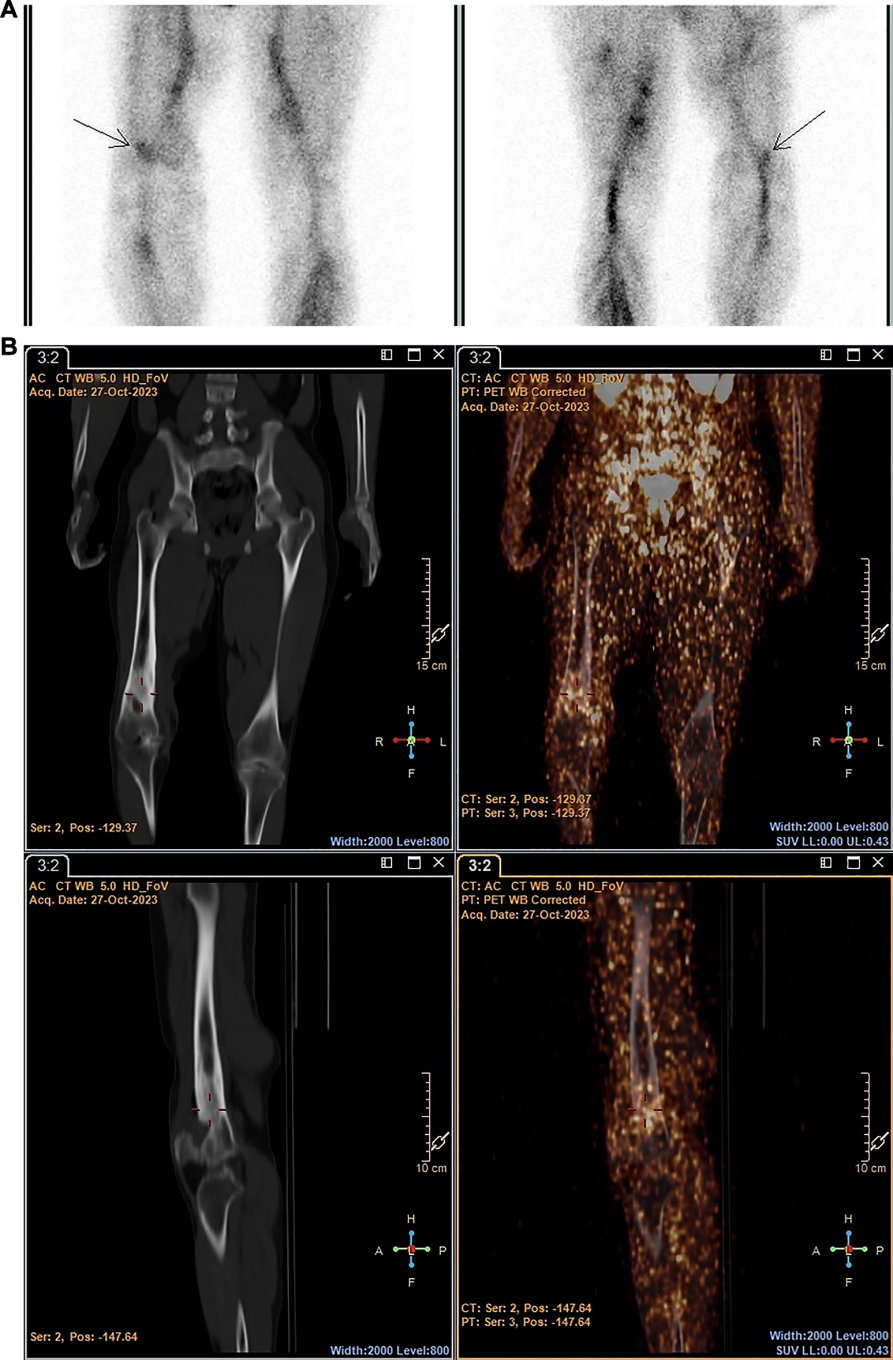
Patient 6. SPECT images obtained with [^99m^Tc]Tc-HMPAO-labelled leukocytes 2 h post injection (**A**) and PET/CT images obtained with ^89^Zr-labelled leukocytes 24 h post injection (**B**) illustrating the accumulation of [^99m^Tc]Tc-HMPAO-labelled leukocytes in the affected site of a patient with chronic femoral osteomyelitis, and minimal accumulation of 89Zr-labelled leukocytes.

The performance of [^99m^Tc]Tc-HMPAO-labelled leukocytes and ^89^Zr-labelled leukocytes, in terms of the ability to accurately detect infection, were similar in two out of seven patients, where both radiopharmaceuticals accumulated in pulmonary oedema due to pneumonia (patient 1) and a hand abscess (patient 4). However, right bronchopneumonia, possibly caused by bacterial infection or reactivated tuberculosis (patient 5), and suspected endocarditis (patient 7) were not detected by either. The latter may have been a true negative result but could not be confirmed. [^99m^Tc]Tc-HMPAO-labelled leukocytes localised in the affected femur of a patient with chronic right femoral osteomyelitis, whereas the ^89^Zr-leukocyte study was non-diagnostic for this patient (patient 6) (see Figure 6).

**Figure 7:**
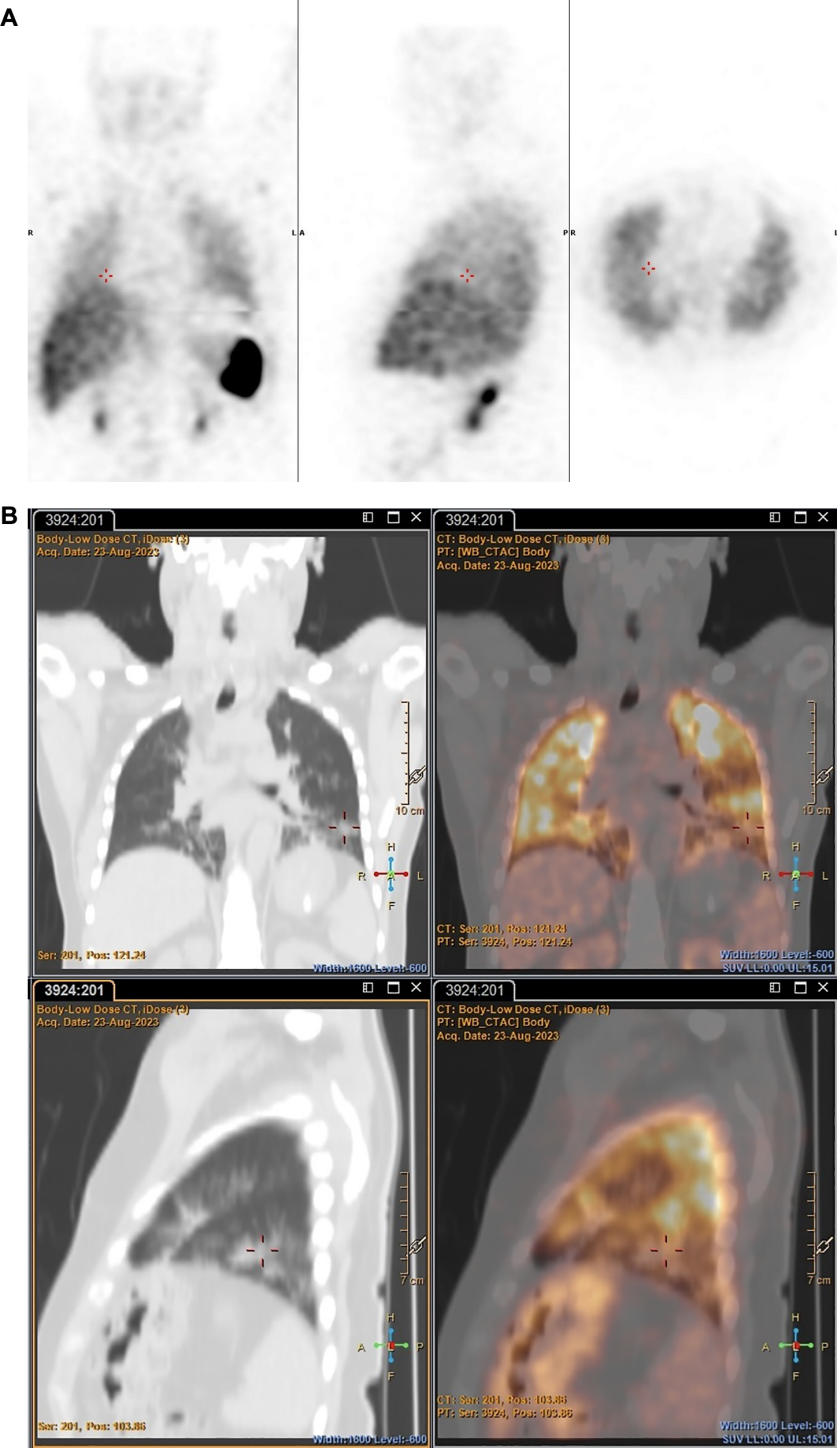
Patient 3. SPECT images obtained with [^99m^Tc]Tc-HMPAO-labelled leukocytes 2 h post injection (**A**) and PET/CT images obtained with ^89^Zr-labelled leukocytes 24 h post injection (**B**), illustrating pathological uptake observed in the left lung with ^89^Zr-labelled leukocytes (PET), but not with [^99m^Tc]Tc-HMPAO-labelled leukocytes (SPECT).

However, ^89^Zr-labelled leukocytes localised in an additional lesion in a patient with bacterial pneumonia (with low diagnostic confidence due to pulmonary cell trapping described above), which was not detected on SPECT (patient 3) (see Figure 7).

**Figure 8:**
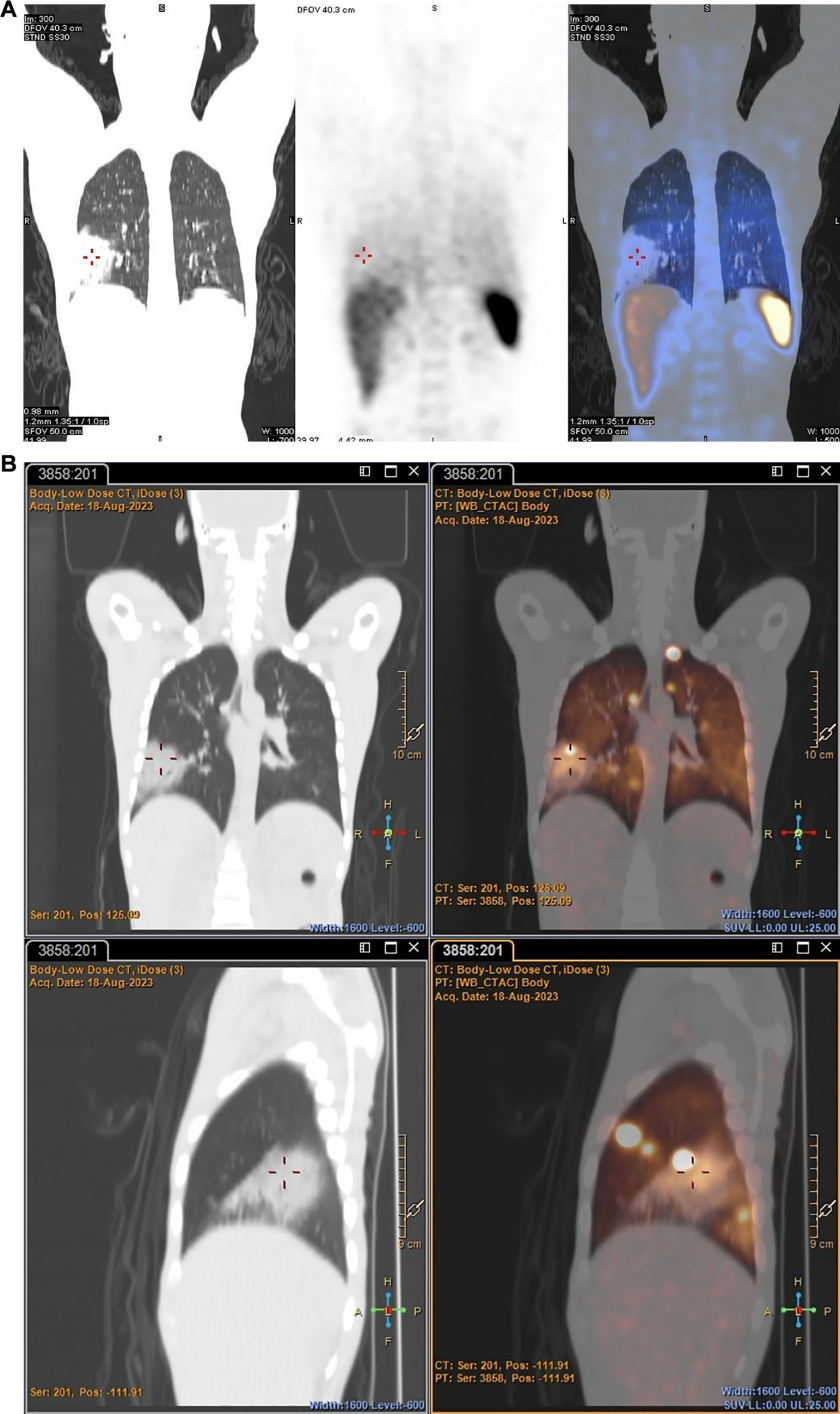
Patient 2. SPECT images obtained with [^99m^Tc]Tc-HMPAO-labelled leukocytes 2 h post injection (**A**) and PET/CT images obtained with ^89^Zr-labelled leukocytes 2 h post injection (**B**), illustrating the increased accumulation of ^89^Zr-labelled leukocytes in a major acute pulmonary lesion.

^89^Zr-labelled leukocytes also illustrated improved pathological uptake in a patient with an acute pulmonary infection (patient 2), which was not evident on SPECT (see Figure 8).

It is worth noting that no skeletal activity was observed in any of the patients imaged in this study, illustrating the *in vivo* stability of the radiolabel and the absence of free [^89^Zr]Zr^4+^.

## Discussion

Infection and the persistent issue of antimicrobial resistance are major contributors to the threat against health and the healthcare system, warranting the search for approaches to diagnose and localise infections earlier and more accurately. Nuclear medicine offers sensitive imaging modalities to detect and localise infection and inflammatory processes at the molecular level and also plays an important role in the monitoring of treatment efficacy (5, 7, 13, 15, 34–37).

Radiolabelled leukocytes for infection and inflammation imaging have some distinct disadvantages and challenges to consider, i.e., the associated risks to the operator when working with blood, maintaining sterility throughout the labelling process, time-consuming preparation, cell viability after labelling, the risk associated with the possible persistence of irradiated cells, and the requirement for two visits to the hospital by the patient. In spite of these challenges, and although many advances have been made in the search for more specific infection imaging agents over recent years, radiolabelled leukocytes remain “gold standard” infection and inflammation imaging agents for various infectious diseases and inflammatory disease processes (2–6). As mentioned previously, ^89^Zr-labelled leukocytes have been a recent topic of interest. Bansal et al. (23) investigated the direct radiolabelling of leukocytes with Df-Bz-NCS and reported a mean radiolabelling efficiency of 22.77% ± 5.02% and high leukocyte viability (90%–95%). Friberger et al. (38) optimised the radiolabelling of human decidual stromal cells (hDSCs), rat bone marrow-derived macrophages (rMac), and human peripheral blood mononuclear cells (hPBMCs) with [^89^Zr]Zr-Df-Bz-NCS and [^89^Zr]Zr-oxine, respectively. The authors achieved relatively high [^89^Zr]Zr-Df-Bz-NCS-cell labelling efficiencies of 71% for hDSCs, 69% for rMac, and 70% for hPBMCs, with no significant decrease in cell viability. They concluded that even though [^89^Zr]Zr-Df-Bz-NCS-cell labelling can be performed under more ambient conditions, [^89^Zr]Zr-oxine-cell labelling outperformed [^89^Zr]Zr-Df-Bz-NCS-cell labelling in terms of stability, cellular retention, and more consistent labelling efficiencies. More recently, the group investigated the *in vivo* biodistribution of hDSCs and rMac radiolabelled with [^89^Zr]Zr-oxine and [^89^Zr]Zr-Df-Bz-NCS, respectively, in healthy rats (39). They obtained labelling efficiencies of approximately 67% for [^89^Zr]Zr-Df-Bz-NCS-hDSCs and 55% for [^89^Zr]Zr-Df-Bz-NCS-rMac with high cell viability (85% and 83%, respectively). The authors reported a significantly increased pulmonary retention of [^89^Zr]Zr-Df-Bz-NCS-labelled cells than [^89^Zr]Zr-oxine-labelled cells for up to 7 days after administration and eventual migration of both tracers to the liver and spleen.

A first-in-human study with [^89^Zr]Zr-oxine-labelled leukocytes in four healthy volunteers was reported in 2022, with promising results (24). Our first-in-human study utilising the cell surface labelling approach, investigated the ability of [^89^Zr]Zr-Df-Bz-NCS-labelled leukocytes to localise sites of infection through PET/CT and compared the scan quality and biodistribution of these labelled leukocytes to the current gold standard, i.e., [^99m^Tc]Tc-HMPAO-labelled leukocytes. Theoretically, ^89^Zr-labelled leukocytes would result in higher-quality images with increased diagnostic value, due to the documented higher resolution and sensitivity of PET when compared to SPECT performed with [^99m^Tc]Tc-HMPAO- and [^111^In]In-oxine-labelled leukocytes in routine clinical practice (6, 23, 40). The long half-life of zirconium-89 makes prolonged/delayed imaging and worldwide shipment of the radionuclide possible. However, in cases where delayed imaging is not required or does not significantly contribute to the clinical investigation, the long half-life can be perceived as a disadvantage due to the unnecessary increased radiation burden to the patient and the need for delayed imaging, which is not a requirement of fluorine-18 (41). The limited availability, resultant high cost and the logistics involved in importing zirconium-89 are further disadvantages (41, 42). To date, zirconium-89 is only produced in certain countries, for example, in the Netherlands and South Korea, and has to be imported into South Africa. Local cyclotron production in South Africa may be possible in the future with the use of yttrium-89 targets (42).

We achieved notable success in the labelling of leukocytes with [^89^Zr]Zr-Df-Bz-NCS, with a significantly higher labelling efficiency (81.7% ± 3.6%; *n *= 8) when compared to Bansal et al. (23), Friberger et al. (38, 39), and our previously published work (31). The improved labelling efficiency obtained in this study may be the result of the shortened [^89^Zr]Zr-Df-Bz-NCS radiosynthesis and therefore greater stability of the complex to allow radiolabelling of cells. Friberger et al. (38) reported a significant decrease in cell labelling efficiency from 69%, when the [^89^Zr]Zr-Df-Bz-NCS complex was utilised for cell labelling 1 h after radiosynthesis, to 31%, when a waiting period of 1.5 h was implemented. Good viability of the [^89^Zr]Zr-Df-Bz-NCS-labelled leukocytes was also observed (88.98% ± 12.51%) that did not illustrate a significant difference from the viability of the [^99m^Tc]Tc-HMPAO-labelled leukocytes (83.12% ± 18.02%). A decrease in the viability of ^89^Zr-labelled cells have been reported, mainly utilising [^89^Zr]Zr-oxine as radiolabel, which can possibly be explained by the position of the radiolabel relative to the cell. [^89^Zr]Zr-oxine crosses the cell membrane and accumulates within the cell, while [^89^Zr]Zr-Df-Bz-NCS binds to the primary amine groups on the cell surface (23, 38, 43). The increased distance of the radiolabel from sensitive intracellular components with the cell membrane labelling approach may suggest decreased radiation exposure and resultant effects of radiation on these components. Similar cell labelling efficiency (approximately 73%) and significantly high viability (approximately 95%) results were reported by Lee et al. (44), who radiolabelled Jurkat/CAR T-cells with [^89^Zr]Zr-Df-Bz-NCS.

Considering the results of the study, we believe our [^89^Zr]Zr-Df-Bz-NCS-leukocyte labelling method is a more robust approach when compared with [^99m^Tc]Tc-HMPAO-leukocyte labelling as it yielded more consistent labelling efficiencies and was less dependent on the leukocyte count of the patient. The only factor that had a significant effect on the ^89^Zr-leukocyte labelling efficiency and yield was the reaction volume, which can easily be optimised in future studies. However, the radiosynthesis of [^89^Zr]Zr-Df-Bz-NCS was slightly more complex when compared to [^99m^Tc]Tc-HMPAO, requiring a skilled and appropriately trained operator for efficient pH adjustment of the [^89^Zr]Zr-oxalate solution without major losses in activity, and accurate pipetting techniques for successful [^89^Zr]Zr-Df-Bz-NCS complexation. The total time required for the preparation of [^89^Zr]Zr-Df-Bz-NCS-labelled leukocytes after leukocyte isolation was also longer (approximately 75 min) than the preparation of [^99m^Tc]Tc-HMPAO-labelled leukocytes (approximately 50 min).

Overall, infection imaging in seven patients with clinically and/or radiologically confirmed infection did not yield convincingly positive results. The non-pathological accumulation of ^89^Zr-labelled leukocytes in the lungs of all seven patients is concerning. The pulmonary passage of intravenously administered cells is a well-known fact and especially reported in studies on stem cell-based therapies. The factors hypothesised to contribute to the pulmonary trapping of intravenously administered cells include cell size and cell adhesion to the vascular endothelium (45–47). Although these factors have been investigated quite extensively, no clear explanation of the mechanisms involved in pulmonary cell trapping are apparent. In the current study, we initially thought cell clumping to be the reason for the focal lung uptake. However, lung uptake still persisted after the introduction of heparin to the dose of [^89^Zr]Zr-Df-Bz-NCS-labelled leukocytes before administration. Further studies to investigate the [^89^Zr]Zr-Df-Bz-NCS label and its possible effect on the adhesion properties of the radiolabelled cell may be recommended. Studies including a wider variety of infections would also contribute to a better understanding of the migration and pulmonary passage of [^89^Zr]Zr-Df-Bz-NCS-labelled leukocytes, as the majority of the patients that could be recruited for this study presented with pulmonary pathology, which could have also contributed to the observed lung trapping phenomenon. Furthermore, immune cells recruited during pulmonary pathology are mostly granulocytes, which were not present in our final radiolabelled leukocyte preparations.

Regarding the whole-body effective dose from [^99m^Tc]Tc-HMPAO-labelled leukocytes in adults, De Vries et al. (32) reported this dose as 0.011 mSv/MBq. The extrapolation of this mean effective dose to the current study to calculate the estimated radiation burden to the patient from [^99m^Tc]Tc-HMPAO-labelled leukocytes resulted in an estimated radiation burden of 2.548–6.532 mSv (mean = 4.524 ± 1.355 mSv). Regarding the whole-body effective dose from zirconium-89, Yoon et al. (28) reviewed recent applications of zirconium-89 in clinical imaging, where most human studies have focused on ^89^Zr labelling of monoclonal antibodies for application in various disease states. The whole-body effective doses reported for these agents were 0.54 mSv/MBq for [^89^Zr]Zr-trastuzumab (dose: 37–74 MBq), 0.61 mSv/MBq for [^89^Zr]Zr-cetuximab (dose: not available), 0.26 mSv/MBq for [^89^Zr]Zr-panitumumab (dose: 37 MBq), 0.38 mSv/MBq for [^89^Zr]Zr-J591 (dose: 37–74 MBq), and 0.41–0.68 mSv/MBq for [^89^Zr]Zr-IAB2M (dose: not available). Yoon et al. (28) concluded that an overall whole-body effective dose of 20–40 mSv for 37–74 MBq of ^89^Zr-labelled monoclonal antibodies can be expected. The extrapolation of this mean effective dose to the current study to calculate the estimated radiation burden to the patient from [^89^Zr]Zr-Df-Bz-NCS-labelled leukocytes resulted in an estimated radiation burden of 16.084–41.019 mSv (mean = 28.469 ± 7.722 mSv).

One clear advantage of [^89^Zr]Zr-Df-Bz-NCS-labelled leukocytes over [^99m^Tc]Tc-HMPAO-labelled leukocytes, which was confirmed in this study, is the long half-life of zirconium-89 and the resultant late time point/longitudinal imaging possible. In the cases where pathological uptake was observed, [^89^Zr]Zr-Df-Bz-NCS-labelled leukocytes illustrated persistent uptake in pulmonary oedema associated with pneumonia at 25.5 h post injection, while the 24 h SPECT images were non-diagnostic. However, the non-specific uptake of ^89^Zr-labelled leukocytes in normal lung parenchyma reduced diagnostic confidence in this case. Accumulation in bacterial pneumonia remained high at 24 h post injection of [^89^Zr]Zr-Df-Bz-NCS-labelled leukocytes, while there was low-grade uptake 18 h after the injection of [^99m^Tc]Tc-HMPAO-labelled leukocytes. Lastly, [^89^Zr]Zr-Df-Bz-NCS-labelled leukocyte accumulation was observed in the hand abscess of patient 4 at 3 h, 26 h, and 75 h after administration, whereas washout of [^99m^Tc]Tc-HMPAO-labelled leukocytes on the delayed images (18 h post injection) was reported. If the pulmonary trapping issue can be addressed, which will result in a higher number of radiolabelled cells available to accumulate in infectious and inflammatory foci, the delayed imaging that is possible with [^89^Zr]Zr-Df-Bz-NCS-labelled leukocytes may illustrate great sensitivity for the localisation of small or difficult to diagnose infectious and inflammatory foci.

We chose to radiolabel mononuclear cells, as this was the approach followed in our *in vitro* and preclinical studies to investigate the role of a specific cell population in infection and inflammation (31). Mononuclear cell isolation from whole blood is also faster and simpler than the isolation of mixed leukocytes through red cell sedimentation and centrifugation. Mononuclear cells are primarily involved in the immune response (48) and can therefore be targeted for infection and inflammation imaging with nuclear medicine. However, in hindsight, the use of a mixed leukocyte population would have been more valuable for infection imaging in our study sample as granulocytes are the major immune cells involved in pulmonary infections (49).

## Conclusion

Successful radiolabelling of human leukocytes with zirconium-89 was achieved utilising Df-Bz-NCS as the bifunctional chelating agent, with our published robust, shortened radiolabelling method. High radiolabelling efficiencies were obtained (81.7% ± 3.6%; *n* = 8) and a mean high viability of the [^89^Zr]Zr-Df-Bz-NCS-labelled leukocytes was also observed (88.98% ± 12.51%). Overall, infection imaging with [^89^Zr]Zr-Df-Bz-NCS-labelled leukocytes did not yield the positive results and high-resolution images that we expected, due to the pulmonary trapping of intravenously administered [^89^Zr]Zr-Df-Bz-NCS-labelled leukocytes. The pulmonary retention of ^89^Zr-labelled cells is a phenomenon that has been observed in published studies and requires systematic investigation to determine the exact cause and variations with different cell types (30, 31, 39). Despite that, generally, similar performance of [^89^Zr]Zr-Df-Bz-NCS-labelled leukocytes to [^99m^Tc]Tc-HMPAO-labelled leukocytes was observed. However, in two cases of pulmonary pathology, [^89^Zr]Zr-Df-Bz-NCS-labelled leukocytes demonstrated potential improved pathological uptake where the accumulation of [^99m^Tc]Tc-HMPAO-labelled leukocytes were unclear or completely absent.

Concerns about the pulmonary trapping of [^89^Zr]Zr-Df-Bz-NCS-labelled leukocytes in all seven patients indicate that further animal work (possibly with primates) is required with other leukocyte types to elucidate this phenomenon and to investigate ways to improve their pulmonary passage for more sensitive imaging. Friberger et al. (39) observed faster lung clearance of [^89^Zr]Zr-oxine-labelled cells than [^89^Zr]Zr-Df-Bz-NCS-labelled cells in an animal model and speculated that the increased and prolonged pulmonary retention of [^89^Zr]Zr-Df-Bz-NCS-labelled cells may be due to higher concentrations of [^89^Zr]Zr-Df-Bz-NCS disrupting cell surface receptors needed for tissue interactions. The results of this study did not demonstrate significant diagnostic superiority of [^89^Zr]Zr-Df-Bz-NCS-labelled leukocytes over [^99m^Tc]Tc-HMPAO-labelled leukocytes. However, larger studies with a wider variety of infectious diseases and utilizing lower concentrations of the [^89^Zr]Zr-Df-Bz-NCS complex may be beneficial. Taking into consideration the cost and logistics involved in the procurement of zirconium-89, irradiation doses to the patient and operator, and the resultant long-lived nuclear waste generated, the focus of further investigations should be on more complicated clinical scenarios where [^99m^Tc]Tc-HMPAO-leukocyte SPECT infection imaging may not be able to accurately detect lesions.

## Data Availability

The raw data supporting the conclusions of this article will be made available by the authors, without undue reservation.
